# Supplementary material for: Regulation of Follicular Development in Chickens: WIF1 Modulates Granulosa Cell Proliferation and Progesterone Synthesis via Wnt/β-Catenin Signaling Pathway
Source: Int J Mol Sci. 2024 Feb 1;25(3):1788. doi: 10.3390/ijms25031788 (PMC10855829; doi:10.3390/ijms25031788)
Supplement: Supplementary file 1 [file ijms-25-01788-s001.zip › Supplemental Figure.pdf]

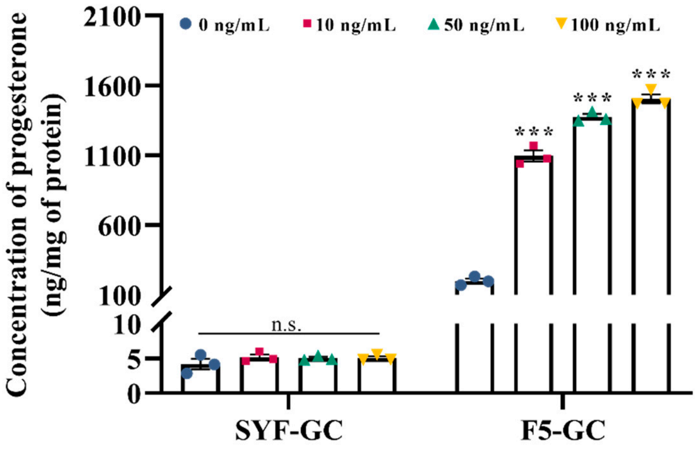

Figure S1. The concentration of progesterone in chicken GC after a 3-hour treatment with FSH.

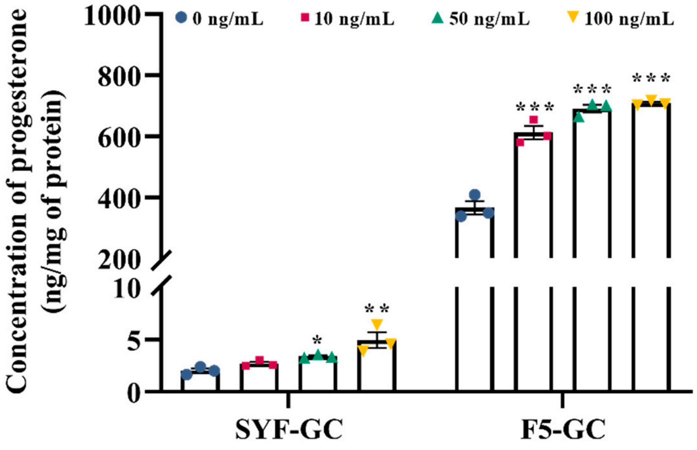

Figure S2. The concentration of progesterone in chicken GC after a 24-hour treatment with FSH.

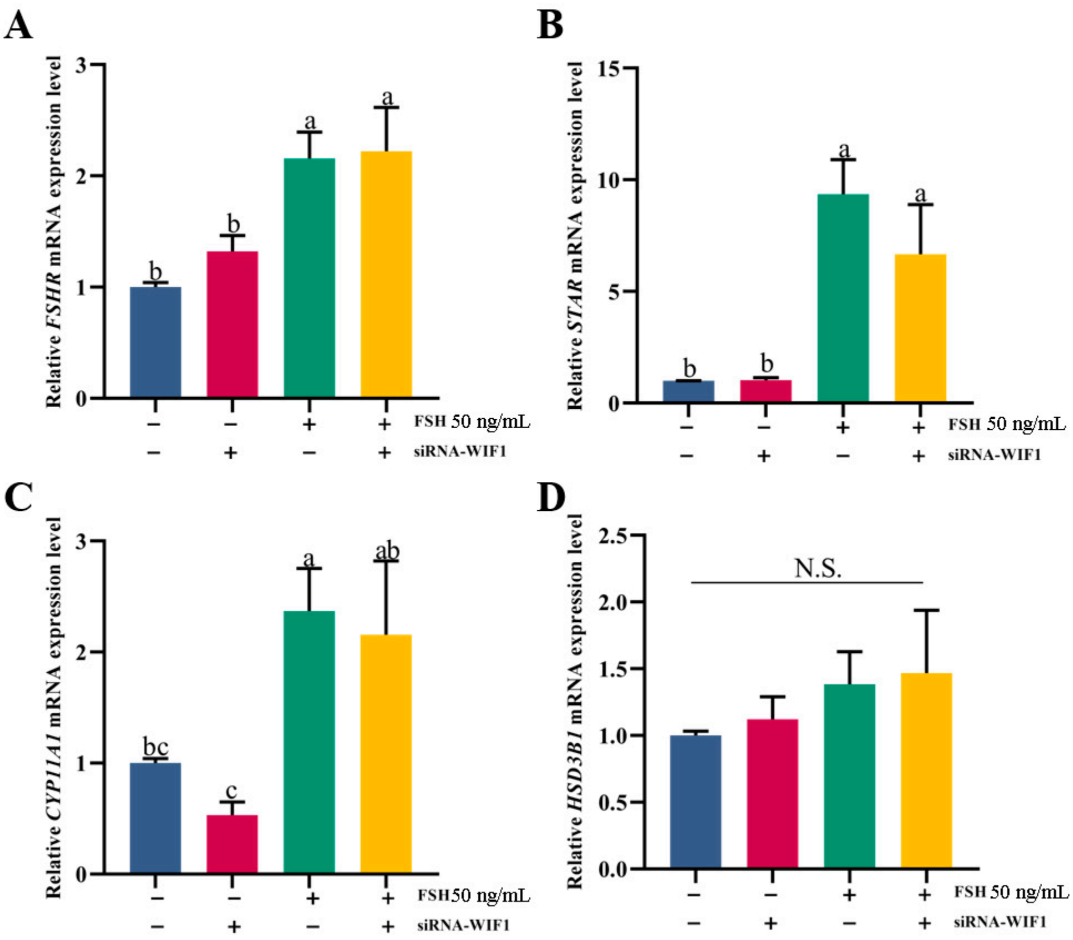

Figure S3. The mRNA expression levels of STAR, CYP11A1, HSD3B1 and FSHR in pre-GC with WIF1 knockdown.

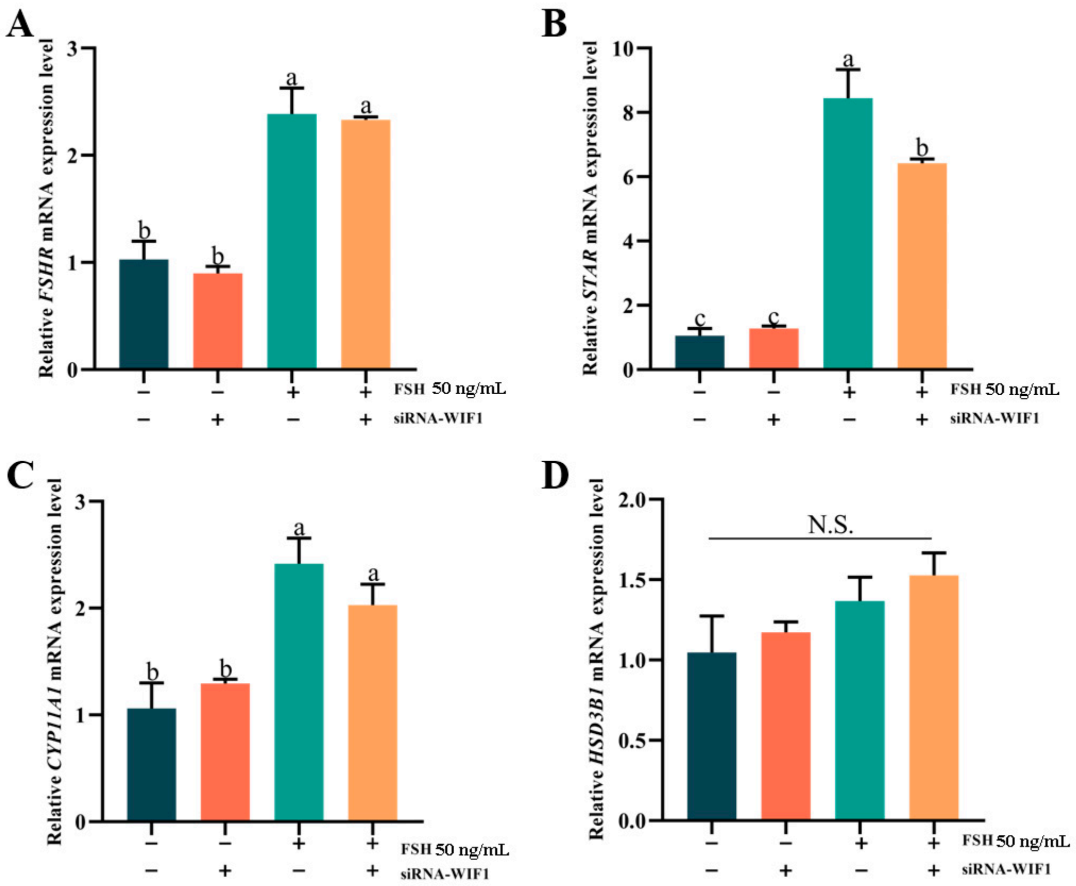

Figure S4. he mRNA expression levels of STAR, CYP11A1, HSD3B1 and FSHR in hie-GC with WIF1 knockdown.
